# Knowledge, attitudes and practices of primary healthcare professionals regarding smoking and smoking cessation among the elderly in France

**DOI:** 10.18332/tpc/173401

**Published:** 2023-10-31

**Authors:** Sylvain Gautier, Anaïs Cloppet, Sarah Mir, Clément Duville, Jean-Manuel Morvillers, Anne-Bérénice Simzac, Katiuska Miliani, Loïc Josseran

**Affiliations:** 1 Département Hospitalier d’Epidémiologie et de Santé Publique, GHU Paris Saclay, Hôpital R. Poincaré, Garches, France; 2 Université de Versailles Saint Quentin, Université Paris Saclay, Montigny le Bretonneux, France; 3 Gérond’if – Le Gérontopôle d’Île-de-France, Paris, France

**Keywords:** elderly, smoking cessation, smoking, addiction, health professionals

## Abstract

**INTRODUCTION:**

Smoking remains a leading cause of preventable death in France, even among the elderly. Although smoking prevalence has decreased overall, it still affects a significant portion of older adults. This study investigates the knowledge, attitudes, and practices of primary healthcare professionals regarding smoking and smoking cessation among the elderly in France.

**METHODS:**

A cross-sectional study involved 300 primary care professionals (general practitioners, pharmacists, nurses) in the Ile-de-France region. Data collection occurred via telephone interviews in September and October 2019. The study employed a questionnaire focusing on knowledge (10 questions), attitudes (12 statements), and clinical practices (7 questions) related to tobacco dependence in older adults. Responses were scored based on correctness for knowledge and appropriateness for attitudes and practices.

**RESULTS:**

The surveyed professionals were predominantly female (57.7%), with a mean age of 53.0 years, and most were non-smokers or former smokers (85.3%). While 66.7% believed older smokers had lower cessation rates, only 64.3% knew it was safe to prescribe nicotine replacement therapy for the elderly. Attitude scores averaged 8.8/12, with pharmacists scoring highest (9.9) and nurses lowest (8.2). Practices scores averaged 2.8/7, with physicians scoring highest (3.8) and pharmacists lowest (1.9).

**CONCLUSIONS:**

Primary healthcare professionals have a relatively good knowledge of the management of tobacco dependence in the elderly and consider it to be part of their mission. However, their confidence in their abilities needs to be strengthened, and many opportunities to counsel and assist this population to quit smoking are still being missed. Preventive approaches to older smokers are essential, in keeping with the concept that ‘every contact with the healthcare system counts’. Improving practice will require education and training that will not only build knowledge but also change perceptions, leading to better attitudes and practices in the management of smoking cessation among older adults.

## INTRODUCTION

In France, despite a decrease in smoking prevalence, smoking remains the leading cause of preventable death, with 75000 smoking-attributable deaths (13% of deaths) in 2015^1,2^. The situation has improved in recent years. Smoking prevalence has decreased from 30.0% in 2000 to 25.3% in 2021, despite a slight rebound observed in 2020^3^. Nevertheless, smoking is evolving differently according to age group: in 2021, its prevalence is estimated at 10.2% among women and 12.4% among men aged 65–75 years. By comparison, these prevalences were close to 5% and 10%, respectively, in 2010, for the same age group^3^. If this prevalence appears low compared to other age groups, it remains high for a population very concerned by its harms. About 70% of the excess mortality related to smoking concerns people aged >60 years^4^: at the age of 70 years, 81% of non-smoking men and 87% of women are still alive, compared to 55% of smoking men and 68% of smoking women. At the age of 80 years, this gap is even more pronounced. Smokers lose at least 10 years of life expectancy compared to non-smokers^5^.

The consequences of smoking go beyond premature mortality. Known to be the main risk factor for cardiovascular disease, chronic obstructive pulmonary disease or cancer, its consequences do not stop there. Smoking is also a risk factor for postoperative mortality in cardiac surgery in the elderly, with a mortality of 14.8% among smokers versus 2.1% among non-smokers (p=0.0001), due to respiratory complications, which are significantly more frequent in smokers^6^. Smoking also accelerates the ageing process and plays a role in pathologies whose prevalence increases with age, such as cognitive decline and dementia^7-9^, osteoporosis^10-12^, age-related macular degeneration^13,14^, and hearing disorders^15^. Thus, smoking compromises health status, leading to disability and impaired quality of life in older people^16-18^. While it is important to quit smoking as early as possible, quitting at any age can significantly prolong life, even after the age of 80 years^19^. Smokers who quit at the age of 35 years gain 6.9 years of life expectancy in men and 6.1 years in women; at the age of 65 years, they gain 2 years in men and 3.7 years in women^20^. Smoking cessation in older adults markedly reduces the risk of cardiovascular events within 1 year of cessation, and the risk continues to decline for many years^21^. The reduction in cancer and COPD mortality is slower but still significant^21,22^. In addition, quitting smoking improves quality of life^23^, and prevents cognitive decline and Alzheimer’s disease^24^.

The majority of elderly smokers started several decades ago and one might think that withdrawal would be more difficult than in younger persons. This is not the case; age is not an obstacle and older smokers seem to have the same chances of achieving long-term abstinence^25-27^. On the other hand, smoking cessation advice from health professionals increases long-term cessation rates and older smokers are responsive to physician advice to quit^28,29^. However, smoking cessation interventions are too rarely offered to older adults^27,30^. The lack of knowledge among health professionals about smoking and cessation in old age, rarely leads to support for elderly smokers^31^.

Despite the benefits and feasibility of smoking cessation in the elderly, no approach in France specifically targets elderly smokers. This study explores the knowledge, attitudes and practices of primary healthcare professionals regarding smoking and smoking cessation among the elderly in France.

## METHODS

### Participants and setting

A cross-sectional study was carried out among 300 primary care professionals (100 general practitioners, 100 pharmacists, 100 registered nurses) in the Ile-de-France region alone (Paris region, France). The representativeness of the sample was ensured by the quota method, with healthcare professionals contacted at random throughout the Ile-de-France region. They were questioned by telephone, using only their professional number, between 23 September and 4 October 2019, by the IFOP polling institute (Institut Français d’Opinion Publique).

### Study instrument

The questionnaire used was developed and adapted from an instrument used in previous studies in the United Kingdom to assess the knowledge, attitudes, and clinical practices of primary care professionals related to tobacco dependence in the elderly. The questionnaire was adapted by a scientific committee composed of public health experts, geriatricians, and representatives of relevant professional associations. Twenty-nine closed and multiple-choice questions, including Likert scales, explore the knowledge (10 questions), attitudes (12 statements) and current clinical practices (7 questions) of professionals regarding tobacco dependence and its treatment in the elderly. The first part assesses the participant’s knowledge with 10 correct or false statements about the prevalence of smoking after the age of 65 years, its consequences, and cessation for this population. Four false statements were included (e.g. there is a risk in prescribing nicotine replacement to an elderly smoker). When the respondent did not know how to answer the question, the response ‘Don’t know’ was recorded. The attitudes of professionals were assessed by asking them to express their agreement with 12 statements on a four-point Likert scale (‘strongly agree’, ‘agree’, ‘disagree’ and ‘strongly disagree’). Clinicians’ practices were assessed using seven questions about reported practices (e.g. how often do you ask elderly patients about their smoking status?). Professionals were asked how often they performed these activities (always, often, rarely, or never). Additional questions were asked about respondents’ sociodemographic characteristics (age, gender, occupation, practice location), current smoking status, and participation in smoking and/or geriatric education (in most cases, these are continuing education courses rather than diplomas).

### Ethics

This telephone survey was conducted in accordance with the World Medical Association Declaration of Helsinki. Informed consent was obtained from all participants. Data were collected and analyzed anonymously and in accordance with the European General Data Protection Regulation.

### Data analysis

Knowledge, attitude, practice, and total scores were calculated from participants’ responses. For knowledge, a correct response scored 1 and an incorrect response scored 0. For attitudes and practices, an appropriate response scored 1 and an inappropriate response scored 0. The appropriate or inappropriate responses were determined by the researchers based on knowledge acquired from the scientific literature and current best practice recommendations. All correspondences have been documented in the Supplementary file. Systematically, for attitudes, the response options ‘agree’ and ‘strongly agree’ were grouped, as were the options ‘disagree’’and ‘strongly disagree’. For practices, the response options ‘always’ and ‘often’ were grouped, as were the options ‘rarely’ and ‘never’. The total score ranged 0–29 and was the sum of all the scores obtained for each question. It was composed as follows: knowledge, 0–10; attitudes, 0–12; and practices, 0–7.

Participants’ characteristics and responses were described using measures of position and dispersion (mean, standard deviation, and range) for numeric variables, and using frequencies and percentages for categorical variables. Chi-squared and ANOVA tests were used to compare respondent characteristics across occupations. Univariate analyses were performed to investigate factors associated with better outcomes. ANOVA and Kruskal-Wallis tests were used for categorical variables with more than 2 terms (e.g. occupation), Student’s t-test and Mann-Whitney tests for categorical variables with 2 terms (e.g. gender), and Pearson and Spearman correlations for numerical variables such as age. Factors were considered statistically significant for p<0.05. Factors associated with better outcomes were also examined in a multivariate analysis using Poisson regression. Results are reported with the incidence rate ratio (IRR) and 95% confidence interval (95% CI). All statistical analyses were performed using R Studio 1.4.1103.

## RESULTS

### Participants

The primary care professionals interviewed were mainly women (57.7%), with a mean age of 53.0 ± 12.3 years, non-smokers or former smokers (85.3%). A minority worked in socially disadvantaged areas (11.6%). Among the 100 physicians, there were as many general practitioners as specialists (51% vs 49%). As for the ex-smokers, 75% had quit smoking more than 5 years ago. On the other hand, 23% of the professionals have followed complementary training, one in gerontology and/or medical tobaccology. Gerontology was the most frequent training (46.4%), followed by tobacco (33.3%) and one-fifth of respondents had taken both courses (20.3%) (Table 1).

**Table 1:** Characteristics of primary healthcare professionals interviewed about their knowledge, attitudes and practices regarding smoking and smoking cessation among people aged >65 years, CAPZEROTABAC Survey, Ile-de-France, 2019

*Characteristics*	*Overall (N=300) n (%)*	*Nurses (N=100) n (%)*	*Pharmacists (N=100) n (%)*	*Physicians (N=100) n (%)*	*p*
**Sex**					<0.001*
Male	127 (42.3)	22 (22)	49 (49)	56 (56)	
Female	173 (57.7)	78 (78)	51 (51)	44 (44)	
**Age** (years), mean (SD) [Range]	**53 (12.3) [25–84]**	**50.1 (9.5) [29–70]**	**47.8 (11.6) [27–69]**	**61 (11.6) [25–84]**	<0.001^†^
**Working in socially disadvantaged area** ^‡^					0.055*
Yes	32 (11.6)	17 (17.9)	7 (7.5)	8 (9)	
**Complementary training**					0.088*
No training	231 (77)	71 (71)	76 (76)	84 (84)	
At least 1 training	69 (23)	29 (29)	24 (24)	16 (16)	
**Tobacco use status**					0.004*
Non-smoker (never smoke or former smoker)	256 (85.3)	76 (76)	92 (92)	88 (88)	
Smoker	44 (14.7)	24 (24)	8 (8)	12 (12)	

* Chi-squared.

† ANOVA.

‡ Information not available for 23 of the professionals interviewed who did not know if they practiced in a socially disadvantage area, i.e. 5 among nurses, 7 among pharmacists and 11 among physicians.

### Total score

The total score ranged from 2 to 28/29. One professional scored 28 and two scored 27. The mean score was 18.4 (median: 19). Nearly 20% of professionals did not achieve the mean score (Table 2).

**Table 2:** Total, knowledge, attitudes and practices scores of primary healthcare professionals interviewed regarding smoking and smoking cessation among people aged >65 years, CAPZEROTABAC Survey, Ile-de-France, 2019 (N=300)

	*Score Mean (SD) Median [Q1;Q3] (Range)*
**Knowledge** (0–10)	6.7 (1.7) 7 [6;8] (1–10)
**Attitudes** (0–12)	8.8 (2.6) 10 [7;11] (1–12)
**Practices** (0–7)	2.8 (2.2) 3 [0;5] (0–7)
**Total** (0–29)	18.4 (4.7) 19 [16;22] (2–28)

### Knowledge score

Two-thirds of professionals (66.7%) thought that older smokers are less likely to quit than younger smokers, which is not true. In addition, only 64.3% knew that it is safe to prescribe nicotine replacement therapy to older adults and 43.3% knew that brief advice is no less effective than more intensive advice in helping older adults to quit smoking.

The knowledge score ranged 1–10 (mean: 6.7) (Table 2). It varied significantly by profession (pharmacists 7.3, nurses 6.6, and doctors 6.2) and age (score decreasing with age, correlation -0.260, p<0.001) (Table 3). These differences were not found in the multivariate model (Table 4).

**Table 3:** Univariate analysis of total, knowledge, attitudes and practices scores of primary healthcare professionals interviewed regarding smoking and smoking cessation among people aged >65 years, CAPZEROTABAC Survey, Ile-de-France, 2019

*Variable*	*Knowledge*		*Attitudes*		*Practices*	
	*mean (SD) [Range]*	*p*	*mean (SD) [Range]*	*p*	*mean (SD) [Range]*	*p*
**Profession**		**<0.001^a^**		**<0.001^b^**		**<0.001**
Nurses	6.6 (1.6) [2–10]		8.2 (2.5) [2–12]		2.7 (2) [0–6]	
Pharmacist	7.3 (1.4) [5–10]		9.9 (1.9) [2–12]		1.9 (1.9) [0–6]	
Physicians	6.2 (1.8) [1–10]		8.4 (3.1) [1–12]		3.8 (2.4) [0–7]	
**Sex**		0.915^c^		**0.026^d^**		0.862
Male	6.7 (1.8) [1–10]		9.2 (2.5) [1–12]		2.9 (2.3) [0–7]	
Female	6.7 (1.6) [1–10]		8.6 (2.7) [1–12]		2.8 (2.2) [0–7]	
**Working in a socially disadvantaged area**		0.688		0.597		0.084
No	6.7 (1.7) [1–10]		8.9 (2.6) [1–12]		2.7 (2.2) [0–7]	
Yes	6.8 (1.4) [3–10]		9.2 (2.3) [2–12]		3.5 (2.2) [0–6]	
**Complementary training**		0.186		**0.004**		**0.008**
No training	6.7 (1.8) [1–10]		8.6 (2.7) [1–12]		2.6 (2.2) [0–7]	
At least one training	6.9 (1.4) [3–10]		9.6 (2.1) [2–12]		3.4 (2.2) [0–7]	
**Tobacco use status**		0.109		**0.026**		0.224
Non-smoker (never smoke or former smoker)	6.8 (1.7) [1–10]		9 (2.6) [1–12]		2.9 (2.3) [0–7]	
Smoker	6.3 (1.7) [2–9]		8 (2.9) [2–12]		2.4 (2.1) [0–6]	
	***Correlation***	***p***	***Correlation***	***p***	***Correlation***	***p***
**Age**	-0.26	**<0.001^e^**	-0.052	0.373^f^	0.119	**0.040**

a ANOVA.

b Kruskal-Wallis.

c Student.

d Mann-Whitney.

e Pearson.

f Spearman.

**Table 4:** Multivariate analysis using Poisson regression of total, knowledge, attitudes and practices scores of primary health care professionals interviewed regarding smoking and smoking cessation among people aged >65 years, CAPZEROTABAC Survey, Ile-de-France, 2019

*Variable*	*Knowledge*	*Attitudes*	*Practices*
	*IRR (95% CI)*	*IRR (95% CI)*	*IRR (95% CI)*
**Profession**			
Nurses (Ref.)	1	1	1
Pharmacist	1.10 (0.98–1.23)	**1.18 (1.06–1.31)**	**0.77 (0.63–0.94)**
Physicians	0.98 (0.86–1.11)	1.04 (0.93–1.17)	**1.62 (1.34–1.96)**
**Age**	0.96 (0.92–1.00)	0.99 (0.96–1.03)	0.96 (0.90–1.03)
**Sex**			
Female	1.00 (0.90–1.10)	0.96 (0.89–1.05)	1.08 (0.93–1.26)
**Working in socially disadvantaged area**			
Yes	1.03 (0.89–1.19)	1.06 (0.94–1.20)	**1.31 (1.06–1.60)**
**Complementary training**			
Yes	1.05 (0.94–1.17)	**1.11 (1.01–1.22)**	**1.41 (1.20–1.65)**
**Tobacco use status**			
Non-smoker (Ref.) (never smoke or former smoker)	1	1	1
Smoker	0.95 (0.83–1.09)	0.93 (0.82–1.05)	0.88 (0.71–1.09)

IRR: incidence rate ratio. a IRR for an increase in age of 10 years.

### Attitudes score

While 82.7% believed they have sufficient knowledge of the benefits of smoking cessation among the elderly to discuss it with them and that 74.3% can easily talk to them about their smoking behavior, only 68.0% of professionals believed they have sufficient knowledge of the therapeutic proposals available for smoking cessation and 61.0% believed that smoking cessation among the elderly falls within the competence of a medical tobaccologist (healthcare professionals who have obtained a diploma attesting to their skills in managing patients who smoke). Finally, 37.7% consider smoking to be one of the few pleasures left to elderly people.

The attitude score ranged 1–12 (mean: 8.8) (Table 2). A significant difference is observed according to the profession (mean score 9.9 for pharmacists, 8.4 for doctors, and 8.2 for nurses). This difference also exists with participation in at least one additional training course (yes 9.6 vs no 8.6), sex (men 9.2 vs women 8.6) and smoking status (ex- or non-smokers 9 vs smokers 8) (Table 3). The multivariate model confirmed the significant difference according to the profession, but only for pharmacists compared with nurses (IRR=1.18; 95% CI: 1.06–1.31), and following the completion of at least one additional training or not (IRR=1.11; 95% CI: 1.01–1.22) (Table 4). Regarding training, it is possible to describe a gradient with a mean score of 8.6/12 for professionals without additional training, 8.8 for those trained in gerontology, 10.2 for those trained in smoking cessation, and 10.7 for those who had validated both.

### Practices score

In current practice, 44.7% regularly asked elderly persons about their smoking status and 35.0% reported it in the clinical file. The motivation of older smokers to quit was regularly assessed by 35.3% of professionals. Brief advice was regularly given by 60.7% of respondents and the benefits of cessation were regularly discussed by only 51.0%. Only 47.7% of professionals regularly provided support during withdrawal. The practice score ranged 0–7 (mean: 2.8), and 26% of respondents had a zero score (Table 2). A significant difference in scores was found according to profession (mean: 3.8 for physicians, 2.7 for nurses, and 1.9 for pharmacists). This difference also appeared with the completion of at least one additional training (3.4 yes vs 2.6 no) and age (score increasing with age, correlation of 0.119, p<0.040) (Table 3). The multivariate model confirmed the impact of the profession on practices for physicians versus nurses (IRR=1.62; 95% CI: 1.34–1.96) and, conversely, for pharmacists versus nurses (IRR=0.77; 95% CI: 0.63–0.94). This difference was also confirmed for the completion of at least one additional training (IRR=1.41; 95% CI: 1.20–1.65). The score changed significantly with exercise in disadvantaged areas (IRR=1.31; 95% CI: 1.06–1.60), mean score 3.5 versus 2.7) (Table 4). Like with the attitudes score, a gradient was observed in the absence of complementary training (2.6); 3.3 for those who attended one training and 4 for those who validated two trainings.

## DISCUSSION

Less than half of primary care health professionals ask their patients aged >65 years about their smoking status, one-third would report it in the medical record and barely more than half of the professionals provide support to older people who want to quit smoking. More than 11% of people aged >65 years smoke daily in France and this prevalence is increasing^3^. Contrary to popular belief, including among the people interviewed in this study, it is not more difficult to quit smoking after 65 years of age^33^. Smoking cessation for older adults is a critical component in promoting healthy ageing. While smoking cessation after the age of 65 years does not strongly reduce the risks associated with smoking, it does rapidly improve the sense of smell and taste independent of age. Smoking cessation also improves respiratory function, including in patients with chronic obstructive pulmonary disease, or reduces insulin doses in diabetic patients^34^. Beyond that, smoking cessation improves the quality of life of elderly smokers even in case of associated chronic diseases. Indeed, Henley et al.^35^ have shown that nearly half of American smokers aged ≥65 years have a chronic disease related to their tobacco consumption and that a quarter of them have cancer also related to this consumption. Given these results, it seems essential that health professionals help older persons to stop smoking.

According to our results, the smoking status of professionals does not seem to affect their attitudes and practices. On the other hand, the high rate of smoking observed in this study, particularly among physicians (12%) and nurses (24%), contrasts with international rates, notably in England where only 5% of physicians are smokers^36,37^. The involvement of a smoking health professional in the management of a smoking patient is less effective than if he or she is a non-smoker^38^. Therefore, it seems important to reduce the prevalence of smoking among health professionals to improve the management of patients who smoke^39-42^.

Smoking cessation in the elderly is no more difficult than for a younger smoker, but it is perceived as more complicated and the responsibility of specialized practitioners by 61.0% of primary care professionals surveyed. This contrasts with the fact that 82% of professionals believe they have sufficient knowledge to help older patients quit smoking. Health professionals’ confidence in their skills and legitimacy needs to be strengthened to better help their patients over 65 years of age. This could be achieved through communication from health authorities to health professionals and continuing education. This difficulty is compounded by a form of fatalism on the grounds that smoking is one of the last pleasures of life^43^, which is not the case, and this fatalism can be countered by the case for improving the quality of life, which remains one of the main priorities of prevention and care, even with advancing age.

### Limitations

This work has several limitations. Firstly, the responses are only declarative. It is therefore possible that for certain questions, the answers are over- or underestimated because the health professionals do not want to be judged (social desirability bias). However, the consistency of the responses between them suggests that this bias remains limited^44^. Second, the type and duration of training were not studied, which may underestimate the impact of training participation on knowledge, attitudes and practices. Finally, this work is based on a sample of 300 respondents (100 per professional category), which may seem limited in relation to the total number of healthcare professionals in the Ile-de-France region. However, Huddlestone et al.^31^ included 150 doctors for an entire country like Great Britain. We therefore consider that a sample of 100 healthcare professionals in each category for a single region, drawn by a professional survey institute with a robust methodology, would be likely to reduce the representativeness bias. However, to the best of our knowledge, this is the first French study of the management of tobacco dependence in people aged over 65 years, by primary care health professionals.

## CONCLUSIONS

Primary healthcare professionals have a relatively good knowledge of the management of tobacco dependence in the elderly and consider it to be part of their mission. However, their confidence in their abilities needs to be strengthened and many opportunities to counsel and assist this population to quit smoking are still being missed. Preventive approaches to older smokers are essential, in keeping with the concept that ‘every contact with the health care system counts’. Improving practice will require education and training that will not only build knowledge but also change perceptions leading to better attitudes and practices in the management of smoking cessation among older adults.

## Data Availability

The data supporting this research are available from the authors on reasonable request.
