# Supplementary material for: Knowledge, attitudes and practices of primary healthcare professionals regarding smoking and smoking cessation among the elderly in France
Source: Tob Prev Cessat. 2023 Oct 31;9:32. doi: 10.18332/tpc/173401 (PMC10616976; doi:10.18332/tpc/173401)
Supplement: Supplementary file 1 [file TPC-9-32-s1.pdf]

Supplementary 1: List of questionnaire questions, categorized by knowledge, attitude, and practice, response options, and corresponding score.

Question	Response options	Corresponding score
**Knowledge**		
In France, approximately 10% of adults over the age of 65 are smokers.	True	1
	False	0
Smokers, on average, lose 10 years of life expectancy.	True	1
	False	0
Smoking can reduce the effectiveness of certain common treatments in older individuals.	True	1
	False	0
Smoking can slow down wound healing in older individuals.	True	1
	False	0
The risk of a heart attack or stroke increases within 24 hours of quitting smoking in an elderly person.	True	0
	False	1
Smoking can accelerate cognitive decline in older individuals.	True	1
	False	0
There is a risk associated with offering nicotine replacement therapy to an elderly person.	True	0
	False	1
Smoking can lead to serious complications in elderly individuals with diabetes.	True	1
	False	0
Older smokers are less likely to quit smoking compared to younger smokers.	True	0
	False	1
Brief smoking cessation advice is less effective than more intensive counselling in helping older individuals quit smoking.	True	0
	False	1
**Attitude**		
I believe it is the responsibility of healthcare professionals to encourage elderly individuals who smoke to quit smoking.	Strongly agree	1
	Agree	1
	Disagree	0
	Strongly disagree	0
I believe I have sufficient knowledge of the harmful effects of smoking in the elderly to discuss it effectively with these patients.	Strongly agree	1
	Agree	1
	Disagree	0
	Strongly disagree	0
I believe I have sufficient knowledge of the benefits of smoking cessation in the elderly to discuss it with these patients.	Strongly agree	1
	Agree	1
	Disagree	0
	Strongly disagree	0
I believe I have sufficient knowledge of the difficulties that can deter elderly individuals from quitting smoking.	Strongly agree	1
	Agree	1
	Disagree	0
	Strongly disagree	0
I believe I have sufficient knowledge of nicotine addiction and withdrawal to discuss it with the elderly patients concerned.	Strongly agree	1
	Agree	1
	Disagree	0
	Strongly disagree	0
I believe I have the necessary skills to encourage elderly smoking patients to quit.	Strongly agree	1
	Agree	1
	Disagree	0
	Strongly disagree	0
I have sufficient knowledge of available therapeutic options that can help elderly smokers quit.	Strongly agree	1
	Agree	1
	Disagree	0
	Strongly disagree	0

Question	Response options	Corresponding score
I am capable of discussing this with these patients (referring to the previous statement).	Strongly agree	1
	Agree	1
	Disagree	0
	Strongly disagree	0
I easily engage in conversations with my older patients about their tobacco use.	Strongly agree	1
	Agree	1
	Disagree	0
	Strongly disagree	0
Smoking is one of the few pleasures that elderly individuals have access to.	Strongly agree	0
	Agree	0
	Disagree	1
	Strongly disagree	1
Quitting smoking is likely to benefit elderly individuals.	Strongly agree	1
	Agree	1
	Disagree	0
	Strongly disagree	0
I believe that genuine support for smoking cessation in elderly patients requires a specialized practitioner.	Strongly agree	0
	Agree	0
	Disagree	1
	Strongly disagree	1
**Practice**		
How often do you ask elderly patients about their smoking status?	Always	1
	Often	1
	Rarely	0
	Never	0
How often do you document the smoking status of elderly smokers in their medical records?	Always	1
	Often	1
	Rarely	0
	Never	0
How often do you assess the motivation of elderly smokers to quit smoking?	Always	1
	Often	1
	Rarely	0
	Never	0
How often do you provide brief advice or encouragement to elderly smokers to consider quitting smoking?	Always	1
	Often	1
	Rarely	0
	Never	0
How often do you discuss the benefits of smoking cessation with elderly smokers?	Always	1
	Often	1
	Rarely	0
	Never	0
How often do you provide support to elderly smokers (including nicotine replacement therapy or specialized devices) to quit smoking?	Always	1
	Often	1
	Rarely	0
	Never	0
Do you ask elderly patients if they smoke cannabis?	Always	1
	Often	1
	Rarely	0
	Never	0

© 2023 Gautier S. et al.
